# Autistic traits, emotion regulation, and sensory sensitivities in children and adults with Misophonia

**DOI:** 10.1007/s10803-022-05623-x

**Published:** 2022-06-21

**Authors:** L. J. Rinaldi, J. Simner, S. Koursarou, J. Ward

**Affiliations:** School of Psychology, University of Sussex, Brighton, UK

**Keywords:** Misophonia, Sound-sensitivity, Sensory sensitivity, Aversion

## Abstract

Misophonia is an unusually strong aversion to everyday sounds such as chewing, crunching, or breathing. Previous studies have suggested that rates of autism might be elevated in misophonia, and here we examine this claim in detail. We present a comprehensive review of the relevant literature, and two empirical studies examining children and adults with misophonia. We tested 142 children and 379 adults for traits associated with autism (i.e., attention-to-detail, attention-switching, social processing, communication, imagination, emotion regulation, and sensory sensitivity across multiple domains). Our data show that autistic traits are indeed elevated in misophonics compared to controls. We discuss our findings in relation to models of the interface between autism, sensory sensitivities, and the specific features of misophonia.

Misophonia is a condition characterised by unusually strong aversions to everyday sounds, such as chewing, crunching, or breathing. Aversive sounds are not particularly loud, and tend to be human bodily sounds from the mouth and nose, but can also be other noises such as repetitive tapping or clicking. Although most people can easily ignore these sounds, people with misophonia experience strong emotional reactions such as anger, disgust, panic and rage, often accompanied by an autonomic response (e.g., increased heart rate; (Dozier et al., 2017; Swedo et al., 2022). Misophonia can be profoundly detrimental to daily life, negatively impacting on family dynamics, school and work-life (Wu et al., 2014). In this study we investigate what traits might contribute to the profile of people with misophonia, looking particularly at traits typically associated with autism. Our aim is to establish whether people with misophonia (henceforth misophonics) show unusual profiles beyond sound aversions themselves. Such data can provide valuable insight into potential underlying mechanisms for the condition. We begin with a brief introduction to misophonia, then describe previous investigations linked to autism, and theoretical arguments for why autism may be an area of interest for researchers of misophonia. We then present two studies testing both adults and children with misophonia, to better understand how autism and autistic traits might play a role in the emergence of misophonia.

Misophonia tends to develop in childhood or adolescence (Rouw & Erfanian, 2018) but can also appear (or intensify) in later life (Cavanna & Seri, 2015). The condition was first recognised two decades ago (Jastreboff & Jastreboff, 2001), and a growing body of research has shed new light on its phenomenology and related characteristics. Several studies have shown differences in people with misophonia beyond sound-sensitivities themselves, including traits of perfectionism, depression, anxiety sensitivity, and obsessive compulsive disorder (Cusack et al., 2018; Eijsker et al., 2019; Jager et al., 2020). Importantly, a number of these traits are known co-morbidities in autism spectrum conditions (henceforth autism; Greenaway & Howlin 2010; Meier et al., 2015). And indeed, over-responsiveness to auditory stimuli is itself a feature of autism (*Diagnostic and Statistical Manual of Mental Disorders*; American Psychiatric Association, 2013). Importantly however, the nature of sound sensitivities in autism are often described as rather different to misophonia. Researchers of autism-linked sensitivities have tended to focus on sounds that are loud, or high, or sudden/unexpected (e.g., (Robertson & Simmons, 2013; Tavassoli et al., 2014) and this is notably different to the sounds of misophonia (i.e., often gentle or ambient sounds from specific categories, such as eating noises). As such, it is still unclear whether misophonia overlaps with autism. One recent review has speculatively proposed that misophonia may potentially account for at least some portion of the sound intolerances found in autistic people (Williams et al., 2021). We therefore investigate these links empirically in the current paper, beginning with a review of how autism has been mentioned in misophonia literature to date.

There have been several case studies of individuals with both autism and misophonia (Haq et al., 2020; Webber et al., 2014) as well as several studies that have attempted to draw a specific link between misophonia and autism in a more direct way. Jager et al., (2020) administered the Autism Spectrum Quotient (AQ; Baron-Cohen et al., 2001) to 575 confirmed misophonics. This widely-recognised measure produces an overall score where 32 + suggests clinically significant levels of autistic traits. However, Jager et al. found no obvious links with autism: their misophonic participants had relatively typical AQ scores, and typical rates of autism for their country of testing (i.e., their mean AQ was 19.3, with 2.4% autism, and this can be compared to baselines of 16.4 and 1.4% respectively; Baron-Cohen et al., 2001b; van der Gaag 2018). Crucially, however, there were no controls, and a number of potential misophonics were removed a priori from their study, specifically if they showed evidence of autism. This makes it impossible to interpret autism/AQ data within this sample, because it has already been stripped of people with autism (Williams et al., 2021). In a second study, Claiborn et al., (2020) questioned 1061 self-declared misophonics and found that 38 (3.6%) reported autism. This figure is slightly higher than the comparable general population (e.g., 2.2% in the USA; Dietz et al., 2020; Williams et al., 2021) but we suggest the wording of their question may have inflated the estimate (*Have you been diagnosed with autism, or do you think you might have it*? [our emphasis]). Nonetheless, even if this figure were a reliable estimate of diagnosis, small numbers make it difficult to interpret co-morbidity when dealing with rare conditions (autism) within rare conditions (misophonia). Hence any such attempt may be stymied from the outset.

There are, nonetheless, a number of reasons to suspect that rates of autism may indeed be higher in misophonia.Williams et al., (2021) present a helpful review describing a number of studies and first-person accounts where sound intolerances reported by autistic people appear to resonate with definitions of misophonia (e.g., strong negative emotional reactions from typical misophonia triggers such as chewing, breathing and snoring). But of course, people with autism might experience misophonia at rates found elsewhere in the population, without any meaningful co-morbidity. Nonetheless, clues to whether we might expect a link with autism also come from the neurological basis of misophonia. For people with misophonia, trigger-sounds are associated with increased activation in anterior insular cortex (Kumar et al., 2017; Schroder et al., 2019), within the brain’s “salience network” (Menon and Uddin, 2010; Uddin 2015). Kumar et al., (2017) also showed increased functional connectivity within the anterior insula during the presentation of misophonic triggers, particularly between the insula and ventromedial prefrontal cortex, posteromedial cortex, hippocampus and amygdala; i.e., limbic regions involved in emotional processing and regulation. This involvement of the limbic system and ‘salience network’ suggests that sounds may be particularly salient and emotionally laden for people with misophonia.Williams et al., (2021) have interpreted these neuroimaging findings in the context of autism. The insula and salience network have been especially implicated in the pathology of autism (Nomi et al., 2019; Uddin, 2015), so Williams et al. hypothesise that such differences in the insula may also potentially increase rates of misophonia in autistic people. They also point out that increased resting-state connectivity between the salience network and amygdala has been suggested in other cases of sensory problems in children with autism (Green et al., 2016; Green & Wood, 2019).

Here we investigate this hypothesis directly, seeking empirical evidence linking autism and misophonia in samples of adults and children. We will administer the AQ questionnaire to both adults and children (these latter via the parent-completed version; Baron-Cohen et al., 2006). If there is a meaningful underlying co-morbidity between misophonia and autism, we predict stronger autistic traits in adults with misophonia, with this difference potentially evident already in children. A small number of previous studies of misophonia have already suggested there may be at least one trait linked with autism, which is heightened attention-to-detail, i.e., the ability to allocate cognitive resources to all details of a task or environment, no matter how small (Young et al., 1989). For example, one study of auditory-evoked potentials found attentional differences between misophonics and controls in their N1 peak, a feature linked to early attentional processing (Näätänen, 1992; Rinne et al., 2006). Another study using the “stop signal task” (which measures response inhibition) suggested that misophonics employed more attentional resources than controls (Eijsker et al., 2019). Finally, in our own earlier study, adults with misophonia were given the Attention-to-Detail subscale of the AQ, and produced significantly higher scores compared to controls without misophonia (Simner et al., 2022). In the current study we will administer the entire AQ, to examine whether misophonics score higher across *all* sub-scales linked to autism.

We will also investigate a second feature associated with autism, which is *emotional reactivity*, a trait linked with *emotion dysregulation*. Emotional reactivity refers to how we respond to any given situation in terms of our emotion-related behaviours, affect and physiology. This trait varies among individuals, and can be measured according to our emotions’ intensity, duration, and onset (i.e., the level of stimulus needed to activate the emotion; e.g., Nock et al., 2008) Emotional reactivity is kept in check by our emotion regulation system, and failures in regulation (i.e., emotional *dys*regulation) can lead to high levels of emotional reactivity, and poorer life outcomes (Grosse Rueschkamp et al., 2018; Nock et al., 2008). Emotional reactivity in children has been linked to clinical conditions in later life (McLaughlin et al., 2010). Importantly, emotion dysregulation is also found in autism (Mazefsky et al., 2013) and more recently in adults with misophonia. Emotion dysregulation, which can manifest as angry outbursts (Giesbrecht et al., 2010) has even been assumed in misophonia simply from evidence of co-morbidities such as depression (Erfanian et al., 2018). Recently, Cassiello-Robbins et al., (2020) showed that difficulties with emotion regulation are positively correlated with symptoms of misophonia in adults, although it is unclear whether these effects reach back into childhood. Here, we take a direct approach in administering a targeted questionnaire to children with and without misophonia, tapping into the three components of emotion dysregulation (i.e., using the Perth Emotional Reactivity Scale, PERS; Becerra et al., 2019) If emotional dysregulation is expected within populations higher in autistic traits, we predict that children with misophonia may score higher on direct tests, such as the PERS.

We also look at one final autism-related trait which is sensory sensitivity (Kern et al., 2007; Robertson & Simmons, 2013; Watling et al., 2001). Sensory sensitivity is characterised by both hyper-sensitivity/ sensory overload (e.g., finding strong smells overwhelming and avoiding them) and hypo-sensitivity/ sensory under-responsivity (e.g., failing to notice strong smells at all and actively seeking them out). Sensory sensitivities can occur in a number of different sense domains (e.g., visual, auditory, olfactory, gustatory, tactile, vestibular, proprioceptive) and an obvious hypothesis is that people with misophonia may well be hyper-sensitive within the auditory domain. As noted above, measures of sensory sensitivity in autism often focus on sounds that are different to the trigger-sounds of misophonia (e.g., asking about problems with the intensity, pitch or suddenness of sounds; e.g., Robertson & Simmons 2013) although a small number of items might be interpreted as applying to misophonia more generically (e.g., finding certain sounds annoying). We therefore ask whether people with misophonia have autism-linked sensory hyper-sensitivity, and look not only at hearing, but also more broadly across multiple sense domains. Where sensitivities arise, they can present early in development (Ben-Sasson et al., 2010) and have a significant negative impact on children’s lives (e.g., in eating, playing, family interactions; Dunn et al., 2016). For all these reasons it is important to recognise whether broad sensory sensitivities are a feature of misophonia in children, and we seek to test this here.

In summary, we propose to test a group of adults and children with and without misophonia (whom we will identify using an appropriate screener; see Methods). We will administer the AQ to adults and children, this latter via parents who will also receive the PERS to measure emotional reactivity (Becerra et al., 2019) and a parent version of the Glasgow Sensory Questionnaire (Robertson & Simmons, 2013) to measure sensory sensitivities. We predict higher rates in all measures, and across both populations, if indeed misophonia is related to autism and to autistic traits.

## Experiment 1

### Methods

#### Participants

We tested 126 misophonic adults (mean age = 30.32 years, SD = 17.21; 92 female/ 25 males/ 5 non-binary/ 4 preferred not to say) and 253 non-misophonic controls (mean age = 20.87 years, SD = 6.35; 199 female/ 47 male/ 3 non-binary/ 4 preferred not to say). We recruited participants from a long-list of self-declared misophonics (recruited via online forums where misophonia is discussed e.g., Facebook, Reddit; Twitter) as well as a general population sample from the University of Sussex community. All participants were screened for misophonia (see below). Those exceeding the diagnostic threshold entered our misophonia group (whether from our self-declared misophonic stream, or general population stream), while those falling below entered our control group. For added conservativeness, we entirely excluded any self-declared misophonic who did not pass the diagnostic threshold, along with participants with incomplete data. Ethical approval was obtained from the local university ethics board prior to testing. Participants took part without monetary incentive, and students were offered course credit.

#### Materials and Procedure

Participants completed our study online, using our in-house web application (www.misophonia-hub.org). Participants were sent a URL via email to take part, which led them directly to our testing page. Participants completed our two measures presented in the order described below (alongside other tests to be reported elsewhere). Our task took 20 min to complete.

*Sussex Misophonia Scale*. This questionnaire is accessed via *The Misophonia Hub* (www.misophonia-hub.org) an online multi-purposes site with resources and tests for people with misophonia. In Part 1 of the questionnaire, participants were asked to indicate whether they hate, or do not mind, a set of 48 misophonia trigger-sounds (e.g., chewing), broken down into eight categories (these categories being; the sound of people eating; the sound of repetitive tapping; the sound of rustling; throat sounds; mouth or nose sounds; voice sounds; background sounds; and also repetitive visual movements -- since these latter are also known triggers of misophonia; Brout et al., 2018). For any *Yes* response, this revealed a full list of triggers within that category. For example, if participants responded *Yes* to *I hate the sound of people eating*, this revealed check boxes for eight types of eating-sound (*crunchy foods; crispy snacks; chewing; lip smacking; swallowing; slurping; wet mouth sounds; other eating sounds*). In Part 2 of the scale, participants were shown 39 statements about behaviours, emotions and outcomes from misophonia, introduced with the question: *How often do these things happen to you?* Examples include: *Hatred of some sounds make me feel lonely* (Item 18); *I want to get pay-back on people who make certain sounds* (Item 37); and *I cover my ears to block out certain sounds* (Item 28). Responses were given on a 5-point scale (*Never*, *Rarely*, *Sometimes*, *Often*, *Always*). The test has been used by several thousand misophonics to date, and its Receiver Operator Characteristics show it to be an “excellent” measure for identifying misophonia in adults (https://psyarxiv.com/5eb39/ see also Rinaldi, Ward, et al., 2022).

*Autism Spectrum Quotient (AQ).* The AQ is a widely used measure for identifying autistic traits (Baron-Cohen et al., 2001). The full questionnaire consists of 50 items divided equally into five subscales measuring different aspects of autism symptomology; i.e., communication (e.g., *I frequently find that I don’t know how to keep a conversation going*), imagination (e.g., *When I’m reading a story, I can easily imagine what the characters might look like*; reversed scored), social skills (e.g., *I find it hard to make new friends*), attention switching (e.g., *I prefer to do things the same way over and over again*) and attention-to-detail (e.g., *I tend to notice details that others do not*). Responses are given on a 4-point Likert scale (*Definitely agree*; *Slightly agree*, *Slightly disagree*, *Definitely disagree*) and total scores range between 0 and 50, where higher total scores indicate stronger autistic traits (i.e., poorer attention-switching, imagination, communication, social skills, and greater attention-to-detail). The AQ scale has been used widely within the autism literature and has been shown to have acceptable internal consistency varying from α = 0.63 − 0.78 (Baron-Cohen et al., 2001; Kurita et al., 2005). The 10 items within the Attention-to-Detail subscale have already been administered to a population which overlaps in part with our participant-sample used here, for a paper exploring attentional influences in misophonia (Simner et al., 2022). Nonetheless, data from this sub-scale are included here as one aspect of the different question of autism symptomatology, but we point out that any findings in the Attention sub-scale should not be taken as a replication of this earlier paper.

## Results

**Fig. 1:**
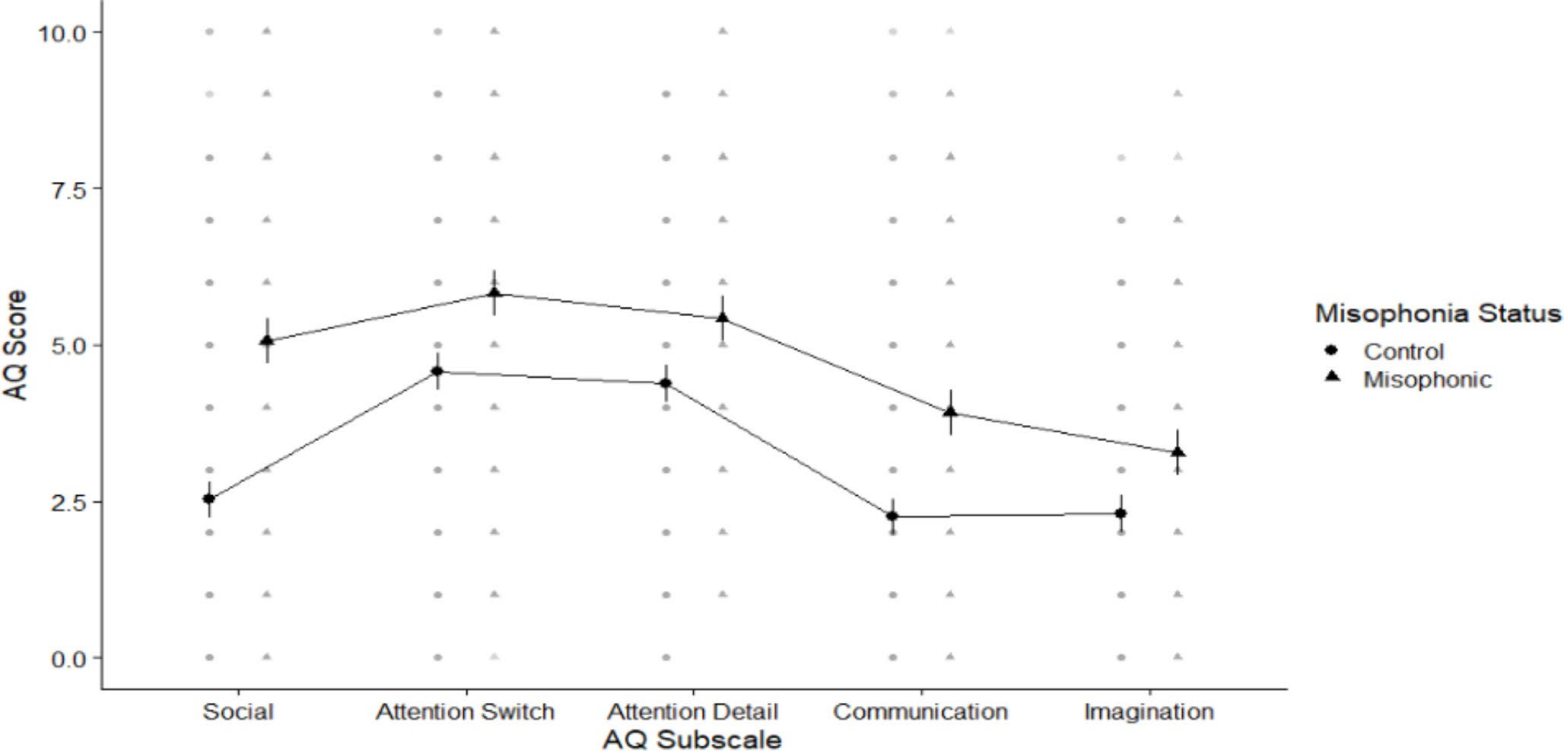
Means plot showing differences between misophonics (shown in triangle) and controls (shown in circles) in each of the AQ subscales from left to right: Social skills, Attention switching, Attention to detail, Communication, and Imagination. Higher scores indicate greater autistic traits

After using the SMS to separate our sample into misophonics and controls (see *Participants*) we then examined scores on the AQ. Within the AQ, scores range between 0 and 50, where higher score indicated greater attention-to-detail. We followed Baron-Cohen et al., (2001) in scoring 1 point for responses of “Slightly/Definitely agree” to positively worded items and “Slightly/Definitely disagree” to negatively worded items. The overall score for adults with misophonia was 24.74 (SD 8.12) compared to 17.29 (SD 6.62) for controls. We explored these AQ data in a 2 × 5 mixed ANOVA crossing group (misophonics vs. controls) with subscale (*Attention to detail, Attention switching, Imagination. Social skills, Communication*). We found a main effect of group (*f* (1, 377) = 91.48, *p* < .001), a less interesting main effect of sub-scale (*f* (3.38, 1273.31) = 111.10, *p* < .001; since scores are generally higher for some sub-scales over others), and a significant interaction (*f* (3.38, 1273.31) = 10.04, *p* < .001)^1^. We explored this interaction using estimated marginal means post-hoc tests which showed that misophonics have a more autistic like profile across all subscales of the AQ (all t-values falling between − 4.04 and − 10.58, and all corrected *p’*s < .001; see Fig. 1) but with largest effect size for Social Skills. All effect sizes were moderate or large (Cohen’s *d* for each sub-scale was: imagination *d* = 0.50, attention to detail *d *= 0.51, attention switching *d* = 0.51, communication *d* = 0.72, social skills *d* = 0.98).

Since scores on the AQ of 32 + are indicative of possible presence of autism, we therefore additionally explored whether significantly more misophonics passed this threshold than controls. We found 27 misophonics (21.4%) scored 32 or above compared to only 7 controls (2.8%), and this difference was highly significant (*χ2* (1) = 33.62, *p* < .001).

## Discussion

We found that a large group of adults with verified misophonia also showed significant differences compared to controls in their autistic traits. Data showed that their scores on the AQ were significantly higher, with differences maintained across all five sub-scales, meaning that people with misophonia showed similar traits to people with autism in having poorer communication, social skills, imagination and attention-switching, but greater attention-to-detail. Largely overlapping data from the sub-scale of attention-to-detail had been presented elsewhere (Simner et al., 2022), but this is the first evidence of an overall scale differences in autistic traits across all five sub-scales. We also found that 21.4% of people with misophonia reached the clinically significant threshold (scoring 32+), compared to just 2.8% of controls. However, we might wish to consider that people with misophonia were recruited by self-referral from websites which might attract particularly strong cases. In other words, we may have found AQ differences by inadvertently recruiting people with the most extreme misophonia (or indeed, individuals who are different in other ways, compared to our controls recruited from the student population for course credit). Hence to address this, we next looked at traits of autism in a group where misophonics and controls were recruited in exactly the same way. In Experiment 2, we screen a large sample of individuals for misophonia, rather than relying on self-referral. At the same time, we extend our investigation into children, to ask whether people with misophonia are already showing differences in autistic traits, at even a young age.

### Experiment 2

#### Participants

We tested 275 participants, comprising 142 adolescents aged 10–14 years (Mean 11.72 SD 1.12; 65 female, 77 male), along with 138 of their parents (116 female, 21 male, 1 prefer not to say) whose children had a mean age 11.72 (SD 1.13; 63 female, 75 male). There were more children than adults since some families ended testing after the child-measures but before the adult-measures. We therefore included these families in our analyses for child-measure only (and we indicate in our *Results* the numbers of participants within each parent-measure). Our adolescent participants will form the basis of our two groups (misophonics vs. controls), which are to be separated during our study using a validated adolescent misophonia questionnaire. (see *Materials and Procedure*).

Our young participants were recruits of the Multisense project (e.g., Rinaldi et al., 2020) a large-scale study focussing on multiple aspects of childhood development, where uptake was 99% of Years 2–5 from 22 junior schools in the south of England, targeted in 2016. The original cohort represented over three thousand children in the initial recruitment wave. The 142 children in our current study were a sub-set of those whose parents had agreed to stay in touch for future screening^2^, and they were tested for the current study four years after initial recruitment. Testing took place between November 2020 and March 2021, and (parent) participants were entered into a £100 prize draw.

#### Materials and Procedure

Participants completed our study remotely, using our in-house web application (www.misophonia-hub.org). Parent participants were sent a URL via email to take part, and this led them directly into our testing page. Our testing battery included the four measures below (alongside other tests to be reported elsewhere). The first two measures were completed by children, taking approximately 15 min, and the last two measures were completed by their parents, taking 15 min.

*SMS-A: Sussex Misophonia Scale for Adolescents (*Rinaldi et al., 2022). This child-completed questionnaire is almost identical to the adult measure described in Experiment 1, with only a single-word difference, substituting the word “work” for “school” in four items (Q12, Q14, Q22, Q31). For example, Item 12 now read as: *I don’t do well at school because of distractions from sounds*. This only very minor change was possible because the scale was originally carefully devised to be adaptable for both adults and children, and has been validated in adolescents with convergent validity against measures of life-satisfaction, quality of life, anxiety, and obsessive compulsive traits (Rinaldi et al., 2022).

*Perth Emotional Reactivity Scale (PERS;* Becerra et al., 2019). The PERS is a 30-item self-report questionnaire measuring the trait of emotional reactivity, as defined by (Davidson, 2010) and (Becerra & Campitelli, 2013). Items measure the ease-of-activation, intensity, and duration of emotions, repeated once for negative emotions (e.g., sadness) and once for positive emotions (e.g., happiness). Responses are given on a 5-point scale from *Very unlike me* to *Very like me*, where higher scores indicate higher reactivity (i.e., faster/ more easily activated emotions, more intense, and longer in their duration). Example items are: *I tend to get happy very easily* (activation-positive emotions), *When I am joyful, I tend to feel it very deeply* (intensity-positive emotions), and *It’s hard for me to recover from frustration* (duration-negative emotions). The PERS has good psychometric properties, with strong loadings of items on their intended factor, concurrent validity with other emotion measures, and good-to-excellent internal reliability (Becerra et al., 2019). Although this test has been used previously on adults, we conducted a linguistic analysis of its vocabulary using age of acquisition norms (Bird et al., 2001; Gilhooly & Logie, 1980) retrieved via the N-Watch psycholinguistics tool (Davis, 2005). This analysis suggested that the vocabulary within this test make it appropriate for adolescents in our study, having a mean age-of-acquisition of approximately 3 years 11 months, with an upper age of approximately 8 years 11 months (based on n66 of its 99 words, which were retrievable from N-Watch). We therefore administered this test to our participants, who were aged 10–14 years.

*Autism Spectrum Quotient for Adolescents (AQ-Adolescent)*. The AQ-Adolescent (Baron-Cohen et al., 2006) has the same items and structure as the adult AQ described in Experiment 1, except that it is completed by a parent/carer, and all first person pronouns are replaced by third person pronouns (e.g., I → S/he [my child]). The AQ-adolescent has excellent test-retest reliability as well as face validity in showing that adolescents diagnosed with autism do indeed score higher than controls (Baron-Cohen et al., 2006).

*The Parent-completed Glasgow Sensory Questionnaire (GSQ-P).* The *GSQ-P* is parent version of the Glasgow Sensory Questionnaire (GSQ; Robertson & Simmons 2013) to assess sensory sensitivities in children. It contains 24 items, covering six sense domains (*visual*, *auditory*, *gustatory*, *olfactory*, *tactile*, *vestibular*) with half of all items measuring *Hyper-sensitivity* (e.g., ‘Does your child ever hate the feeling or texture of certain foods in his/her mouth?’) and half measuring hypo-sensitivity (e.g., ‘Does your child ever complain of having a weak sense of taste?’). There were five possible responses: *Never, Rarely, Sometimes, Often, Always*. The adult measure shows excellent reliability (α = 0.94; GSQ; Robertson & Simmons 2013) and our parent adaptation ensured all items were suitable for parents (e.g., Do you → Does your child).

## Results

### Identifying Children with Misophonia

We applied the required threshold to identify children with misophonia, which were those scoring 49 or higher (out of 156) within the *Sussex Misophonia Scale for Adolescents*, taken from (Rinaldi et al., 2022). The resultant misophonia group comprised 15 children, including 9 girls (mean age 11.67, SD 1.32) and 6 boys (mean age 11.00, SD 0.89). The remaining 127 children were designated controls, and comprised 56 girls (mean age 11.67, SD 1.22) and 71 boys (mean age 11.83, SD 1.03). We then compared our groups in the remaining measures below, where we give participants numbers for each analysis. We ran our analyses in R using the packages “afex” for ANOVA, “emmeans” for post-hoc estimated means tests, and “tidyverse” for general data wrangling.

### Do children with misophonia show greater autism-related traits compared to their peers?

**Fig. 2:**
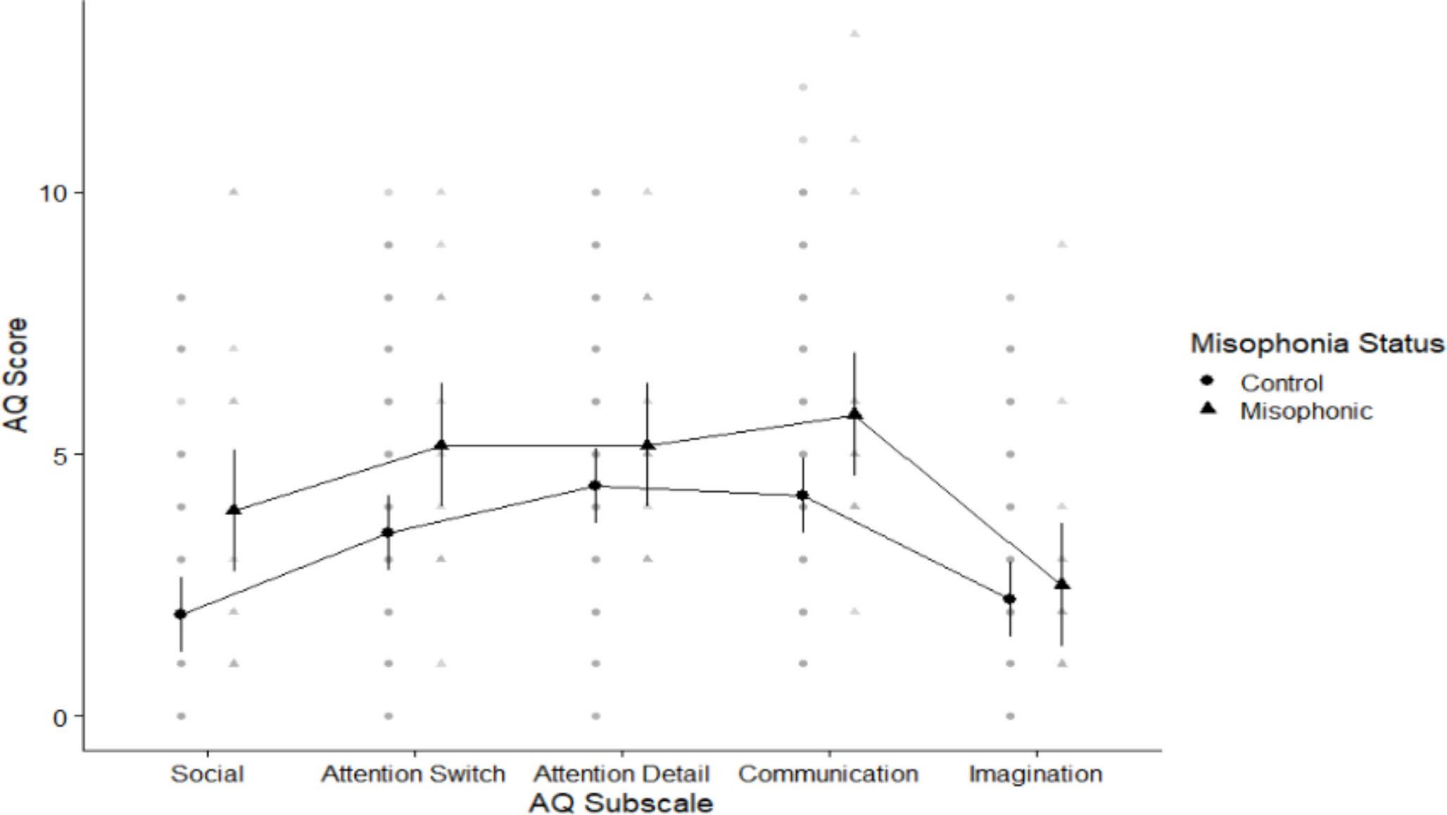
Means plot showing differences between misophonics (shown in triangle) and controls (shown in circles) in each of the AQ subscales from left to right: Social skills, Attention switching, Attention to detail, Communication, and Imagination

We first looked at our parent-report measure for autistic traits (AQ-Adolescent; (Baron-Cohen et al., 2006). The overall score for children with misophonia was 25.00 (SD 1.30) compared to 18.78 (SD 8.85) for controls (based on 12 misophonics and 110 controls). We again explored the AQ in a 2 × 5 mixed ANOVA crossing group (misophonics vs. controls) with subscale (*Attention to detail, Attention switching, Imagination. Social skills, Communication*; see Fig. 2) .We again found a main effect of group (*f* (1, 120) = 5.05, *p* = .027), the less interesting main effect of sub-scale (*f* (3.16, 378.76) = 15.48, *p* < .001; see Experiment 1), and no interaction (*f* (3.16, 378.76) = 1.42, *p* = .234). This suggests children with misophonia scored higher than controls across all sub-scales to a similar degree.

There were no significant differences across groups in the number of participants scoring in the clinically relevant zone of 32+, although potentially due to small numbers (Fishers Exact *p = .616*). Two children with misophonia (out of 12) scored 32 or higher, compared to 11 controls (out of 110). This would represent high percentages (12.5% and 10% respectively) perhaps indicating that even controls in this sample were unusually high in autistic traits. Nonetheless, this makes our target comparisons more, not less, conservative, since even within a sample with apparently higher rates of autistic traits, children with misophonia still stand out as being different to their peers.

### Do children with misophonia show greater emotional reactivity compared to their peers?

**Fig. 3:**
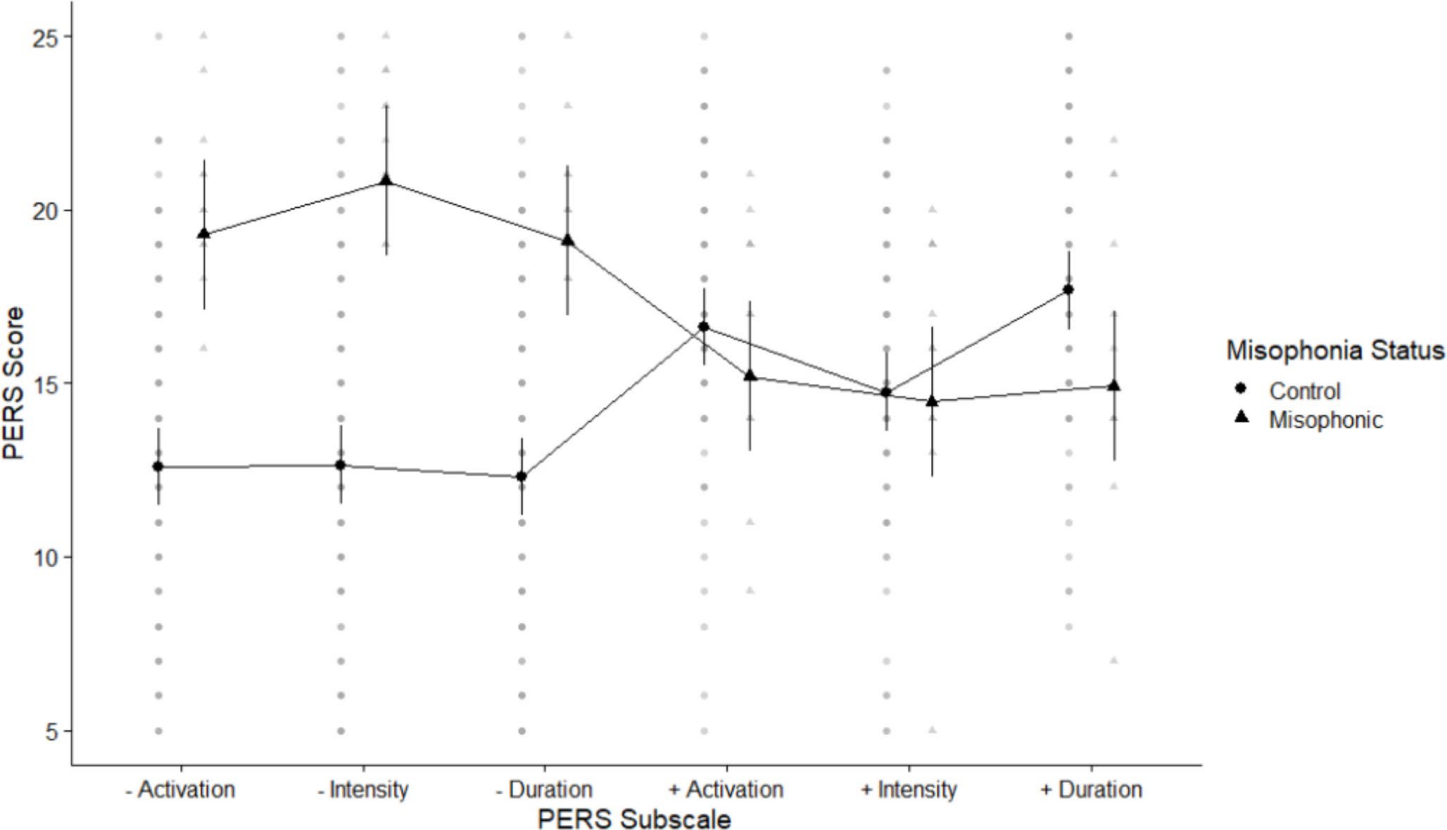
Means plot showing differences between misophonics (shown in triangle) and controls (shown in circles) in each of the PERS subscales. Negative subscales are shown with –, and positive subscales with +

We examined our data for child-reported differences in the PERS (Becerra et al., 2019). Mean scores across the positive-emotion dimensions for children with misophonia were 48.91 (SD 11.92) compared to controls who scored 52.07 (SD 11.14) (based on 12 misophonics and 116 controls). Across the negative-emotion dimensions mean scores for children with misophonia were 62.90 (SD 5.74) compared to controls who scored 41.27 (SD 12.80). We explored these differences using a 2 × 2 × 3 ANOVA crossing group (misophonics, controls) with valence (positive, negative) and subscale (activation, intensity, duration; See Fig. 3). We found a main effect of group and significant interactions between group and both valence and subscale, although we did not find a 3-way interaction, see Table 1. We ran post-hoc estimated marginal means tests to explore the interaction across group and valence (i.e., positive/negative emotions), and found that misophonics were significantly higher than controls across negative scales (*t*(235) = -5.86, *p* < .001) but were no different from controls across positive scales (*t*(235) = -1.21, *p* = .229). We also explored the interaction between group and subscale (i.e., activation/ intensity/ duration) and found significant groupwise differences for all subscales (i.e., misophonics higher than controls) with the subscales of activation and intensity showing the largest effect sizes (activation *t*(0.90) = -2.93, *p* = .004: intensity *t*(0.90) = -4.39, *p* < .001: duration *t*(0.90) = -2.25, *p* = .026).

**Table 1:** ANOVA between group (misophonic, control), valence (positive, negative) and subscale (activation, intensity, duration)

Effect	Degrees of Freedom	Mean Squared Error MSE	*F*	*p*	Effect size *η2G*
Group	1, 119	40.38	12.24 ***	< 0.001	0.038
Valence	1, 119	50.98	0.32	0.573	0.001
Group: Valence	1, 119	50.98	22.35 ***	< 0.001	0.083
Subscale	1.98, 235.05	4.13	0.57	0.563	< 0.001
Group: Subscale	1.98, 235.06	4.13	4.76 **	0.01	0.003
Valence: Subscale	1.99, 236.53	3.18	12.91 ***	< 0.001	0.006
Group: Valence: Subscale	1.99, 236.54	3.18	0.88	0.416	< 0.001

### Do children with misophonia show greater sensory sensitivities compared to their peers?

**Fig. 4:**
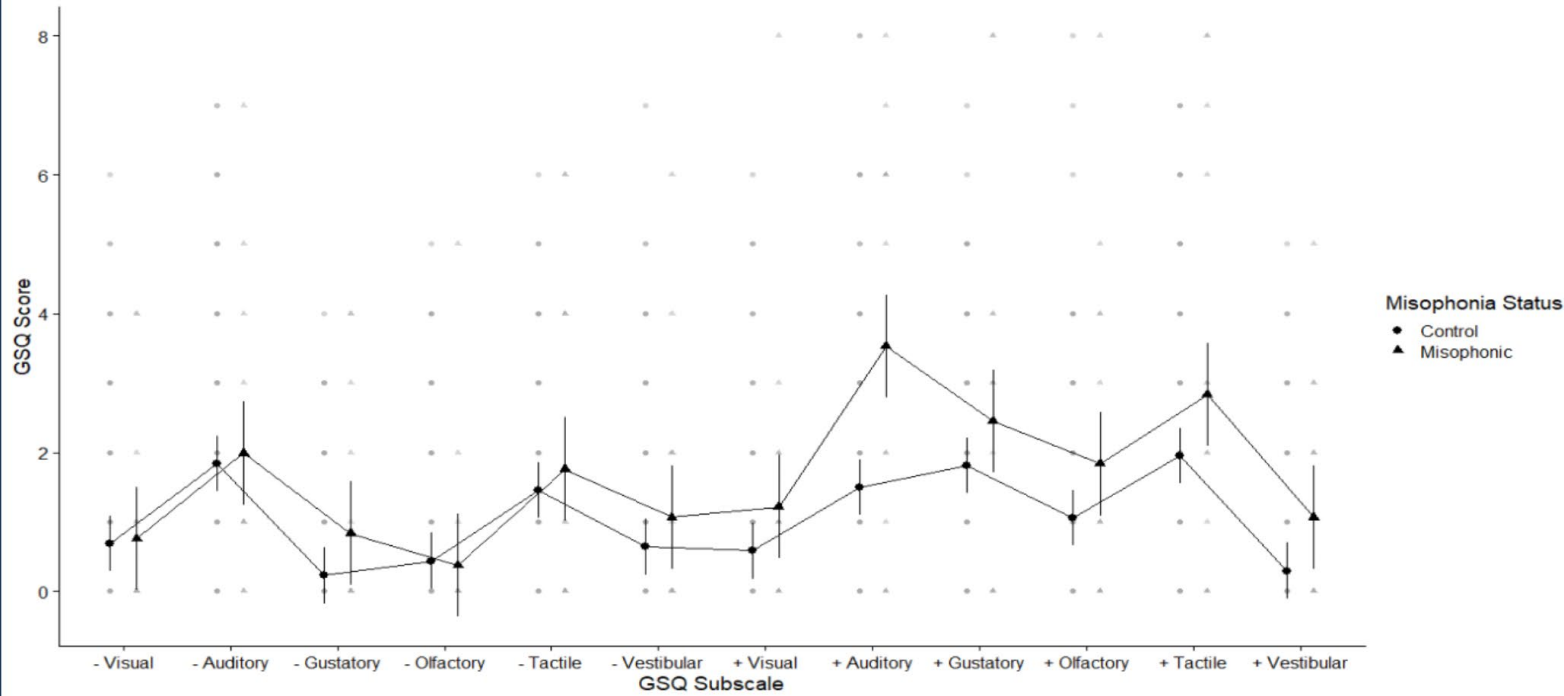
Means plot showing differences between misophonics (shown in triangle) and controls (shown in circles) in each of the GSQ subscales where – is shown for hypo-sensory sensitivities and + is shown for hyper-sensory sensitivities

Finally, we looked at the parent-report measure for sensory sensitivities (GSQ-Parent), where higher scores indicate greater sensitivities. The overall score for children with misophonia was 22.62 (SD 18.65) compared to 15.39 (SD 11.09) for controls (based on 13 misophonics and 119 controls). Figure 4 shows this broken down across conditions. We explored these data using a 2 × 2 × 6 mixed ANOVA crossing group (misophonics, controls) with sensitivity (hypo-sensitive, hyper-sensitive) and sense (Visual, Auditory, Tactile. Gustatory, Olfactory, Vestibular). The outcome of this analysis is shown in Table 2, but can be summarised as follows. First, we found a main effect of sense, simply because people were more sensitive in some senses than others (e.g., the tactile sense more than the visual sense). We also found a main effect of sensitivity (because people showed greater hyper-sensitivity than hypo-sensitivity) as well as an interaction between these two factors (i.e., larger differences between hyper and hypo-sensitivity in some senses over others; cf. gustation vs. vestibular). Importantly however, we found a main effect of group -- since children with misophonia were more sensitive than controls. We also found an interaction between group and sensitivity. We explored this interaction with post-hoc estimated marginal means tests, and found that misophonics were significantly more *hyper*-sensitive than controls (misophonics Mean 2.16 vs. controls Mean 1.20) but they were no difference to controls in *hypo*-sensitivities (hyper, *t* (195) = 2.91, *p =* .004: hypo, *t* (195) =-0.75, *p =* .453; p values are corrected). There was no significant three-way interaction (see Table 2), suggesting no evidence that this hyper-sensitivity of misophonics was mediated by sense (i.e., misophonics were hyper-sensitive across all senses; see Fig. 4).

**Table 2:** ANOVA between group (misophonic, control), sensitivity (hypo-, hyper-) and sense (Visual, Auditory, Gustatory, Olfactory, Vestibular)

Effect	Degrees of Freedom	Mean Squared Error MSE	*F*	*p*	Effect size *η2G*
Group	1, 130	11.98	4.25 *	0.041	0.013
Sense	4.40, 572.59	1.8	23.35 ***	< 0.001	0.046
Group: Sense	4.40, 572.60	1.8	1.06	0.376	0.002
Sensitivity	1, 130	3.19	19.83 ***	< 0.001	0.016
Group*Sensitivity	1, 130	3.19	5.54 *	0.02	0.005
Sense*Sensitivity	4.27, 555.07	1.58	6.84 ***	< 0.001	0.012
Group*Sense*Sensitivity	4.27, 555.08	1.58	1.75	0.133	0.003

## Discussion

Our study examined whether adolescents with misophonia already show autism-related traits, similar to adults (Experiment 1). We found that children 10–14 years with misophonia scored higher in all sub-scales of the AQ, as well as in emotional reactivity to negative (but not positive) emotions, and also showed greater sensory hyper-sensitivities, both within the auditory domain, and more broadly across multiple senses. We discuss the interpretation of these findings in combination with our adult data in the General Discussion below.

### General discussion

Our studies aimed to test the hypothesis that misophonia may be linked to autism in non-trivial ways. We found evidence that both adults and children with misophonia show greater autism-related traits, using the AQ measure (Baron-Cohen et al., 2001, 2006). Our previous study had shown elevated Attention-to-Detail in our adult sample (Simner et al., 2022), but here we show autistic tendencies across all five autistic subscales, including attention-switching, communication, social skills and imagination. In the current study, we also found that this effect is already apparent in children with misophonia aged 10–14 years. We found, too, that these same children were demonstrating greater emotional dysregulation/ reactivity than their peers (Becerra et al., 2019). These data suggest children with misophonia are faster to activate negative moods, feel negative moods more intensely, and maintain negative moods for longer. These data are also compatible somewhat with adult findings (examining different aspects of emotion dysregulation; Cassiello-Robbins et al., 2020) and now show that emotion dysregulation reaches back into childhood. Finally, children with misophonia also demonstrated greater sensory hyper-sensitivity (using a parent version of the Glasgow Sensory Questionnaire; (Robertson & Simmons, 2013) not only in the auditory domain, but also more widely across multiple senses.

To some extent it is no surprise to find sensory hyper-sensitivities in the same group where misophonia has been identified, at least in the auditory domain. The measure used here (i.e., the GSQ adapted for parents) contains two items on auditory hyper-sensitivities, one of which might loosely fit the definition of misophonia, at least in part (*Does your child find certain noises/pitches of sound annoying?*) and a second which characterises a more typical autistic sensitivity to sound (*Does your child dislike loud noises?*) and might signal the related condition of hyperacusis (in which loud noises can cause pain or ‘fullness’ in the ears; Baguley 2003; Baguley & McFerran, 2011). Important here, however, was to examine whether children with misophonia have hyper-sensitivities in other senses. This was indeed the case: we found hypersensitivities across multiple sense domains (auditory, but also tactile, visual etc.) Also important was to establish whether auditory hyper-sensitivity in misophonia was paired with a co-occurring auditory *hypo*-sensitivities. Studies have shown moderate-to-large relationship between hyper- and hypo-sensitivity, in that both hyper and hypo-sensitivities can cluster within a single individual (Sapey-Triomphe et al., 2018). Here we found misophonics showed evidence of higher hyper-sensitivities in multiple senses, but no difference in their hypo-sensitivities compared to controls.

It is important to recognise sensory sensitivities (and indeed other autism related traits), and to recognise them early, because they can have a significant impact on lives. Sensory hyper-sensitivities in children, for example are associated with higher levels of anxiety, shyness and more challenging behaviours (Dunn et al., 2016). Sensory sensitivities have also been linked to dyspraxia (Buitendag & Aronstam, 2010), play preferences (Bundy et al., 2007), compulsive-like behaviour (Dar et al., 2012), stereotyped movements (Gal et al., 2010) and feeding problems (Davis et al., 2013). Our findings suggest that understanding sensory sensitivities – especially as they arise in children -- might be a key focus for misophonia scientists.

Our findings of poorer emotion regulation might also be considered to mirror a core trait associated with misophonia: that negative emotional reactions are triggered more readily, not only specifically in response to sounds, but also more generally in everyday life. It is likely that some of the same neurological underpinnings which cause unusual negative emotions in response to sounds (Kumar et al., 2017; Schröder et al., 2019) may also be at play in the emotion dysregulation found more broadly here. Williams et al., (2021) has speculated a link between autism and misophonia based on their neurological profiles. As in misophonia, the insula and salience network have been especially implicated in autism (Nomi et al., 2019; Uddin, 2015), and indeed, increased resting-state connectivity between the salience network and amygdala has also been found with sensory hyper-sensitivity more broadly (in children with autism; (Green et al., 2016; Green & Wood, 2019). Here we investigated this hypothesis directly, findings links between misophonia and both autism, and sensitivities. In summary, we found one or more differences across all measures administered, and our hypotheses were supported by data from 126 adult and 15 child misophonics, and 391 controls. Although our sample size of child misophonics was small, it still yielded significant and meaningful effect sizes. Moreover, it represents the first ever sample derived from population screening, rather than clinic-referral, and so are particularly valuable in this regard.

Our findings, linking misophonia with autistic traits, are difficult to dismiss as recruitment biases, since although our adults were largely self-referred as misophonic, our children were not. And there were no differences in the recruitment methods between our misophonic and control children, suggesting that differences reside in their classification along the lines of misophonia. Importantly, our screening for misophonia was child-completed, while two of our other measures were parent-completed, meaning our results cannot be dismissed as a response bias (e.g., an acquiescence bias) because our data come from different individuals rating the same child. In summary, our research centred around the idea that dispositional differences might shed light on why people with misophonia find everyday sounds overly aversive. We found elevated autistic traits across all 5 subscales of the AQ, as well as the autism-related traits of sensory sensitivity and emotion dysregulation. It is important to note, however, that misophonia and autism are not equivalent. Only 27 of the 126 adults with misophonia in our study reached the clinically significant threshold for autism in the AQ (i.e., a score of 32 or higher). In other words, people with misophonia need not have autism, just as people with autism need not have misophonia. Nonetheless, it is important to recognise the link we have demonstrated between the two, especially where support can be offered, and perhaps most especially for children.

## Footnotes

1 Our entire sample were 77% female, as is often the case in self-referred cohorts (Dindia & Allen, 1992). However there were no gender differences across groups (*χ2 *= 0.98, *p* = .32). And when we repeated our analyses with gender as a covariate we found no difference in any pattern of results (i.e., same main effects of group and subscale, and interaction between group and subscale, at *ps* < .001).

2 Our sample who participated here were representative of the the original wave across a range of well-being metrics taken at earlier recruitment; i.e., no different in positive affect (*t*(141.95) = 0.70, *p* = .49), negative affect (*t*(141.86) = 1.70, *p* = .09; PANAS-C, (Laurent et al., 1999), pro-social behaviour, emotional symptoms, conduct, hyperactivity or peer problems (i.e., no effect of group (*F*(1,534) = 0.79, *p* = .374), nor interaction (*F*(3.21, 1712.16) = 0.53, *p* = .673; Goodman, 2001)). Our sample were higher than the remainder in spelling (*t*(149.21) =-6.04, *p* < .001) and maths (*t*(113.88) =-7.16, *p* < .001) and this is perhaps to be expected from the children of parents who sign up for continued research (given the heritability of contentiousness and intelligence; Devlin et al., 1997; Luciano et al., 2006). The important point, however, is that our entire sample for the current study were recruited in exactly the same way, and we look *within* this subset, based on a screening for misophonia. In other words, our sampling is unrelated to our findings on misophonia.
